# Single‐Trial Relationships Between the Error‐Related Negativity, Pe, Error‐Related Pupillary Dilation Response, and Post‐Error Behavior

**DOI:** 10.1111/psyp.70216

**Published:** 2026-01-09

**Authors:** Sara LoTemplio, Jack Silcox, David L. Strayer, Brennan R. Payne

**Affiliations:** ^1^ Rubenstein School of Natural Resources University of Vermont Burlington Vermont USA; ^2^ Department of Psychology University of Utah Salt Lake City Utah USA; ^3^ Interdepartmental Neuroscience Program University of Utah Salt Lake City Utah USA

**Keywords:** ERN, PDR, Pe, PES, post‐error adjustment

## Abstract

The amplitude of the error‐related negativity (ERN) is known to be correlated with attention to task and general cognitive control abilities. However, previous research has struggled to consistently link ERN amplitude with behavioral accuracy or reaction time in the task from which the ERN is being measured. This lack of relationship could be due to many factors that are difficult to control for, so explorations of other converging measures to understand error‐processing and subsequent behavior adjustment are warranted. The current study examines how two other physiological markers of error‐processing—the phasic pupillary dilation response (PDR) and the positivity following an error (Pe)—relate to post‐error behavior. Additionally, we also examine relationships between the three physiological indices of error‐processing. In the study, EEG and pupillometry were simultaneously recorded while participants completed 24 blocks (50 trials each) of an Ericksen Flanker task. For post‐error accuracy, we found that on a single‐trial level, the amplitude of all three physiological error‐processing indices for error trials predicted post‐error accuracy. At the subject level, only the PDR predicted average post‐error accuracy. For post‐error slowing, at the single‐trial level, only the Pe predicted post‐error slowing, whereas only the ERN predicted post‐error slowing at the subject level. We also found that both the ERN and Pe correlated with PDR amplitude. This is consistent with our hypothesis that the Pe and PDR may share underlying neural mechanisms, but qualified by the fact that the ERN, which is not hypothesized to have shared neural mechanisms, also predicted unique variance in pupillary amplitude. Collectively, these results suggest that the PDR and Pe might represent promising indicators of post‐error behavior adjustment and highlight the need to examine relationships at multiple levels of analysis.

## Impact statement

While the ERN response occurs after an error, it has been difficult to link its amplitude to behavior adjustment following the error. The current study examines how two other physiological markers of error‐processing, the Pe and the PDR, affect behavior adjustment. Importantly, we compare relationships at both the trial and subject levels. To our knowledge, no study has directly compared brain‐behavior relationships of all physiological error markers at both levels of analysis in the same sample.

## 1 Background and Significance

To achieve the best performance on the task, it is usually helpful to detect when we make mistakes—presumably so that we can then correct them and get back on track. However, how this process unfolds in the brain remains vexingly elusive. One of the most common ways to measure error‐processing in the brain is through electroencephalography (EEG), including the event‐related potential (ERP) method. Specifically, the error‐related negativity (ERN), a negative deflection in the EEG waveform that occurs after the commission of a motor error (for review see Gehring et al. 2012), has received considerable attention in recent years. The amplitude of the ERN is known to be correlated with attention to task, as well as general cognitive control abilities (for review see Meyer and Hajcak 2019). However, previous research has struggled to consistently link ERN amplitude with behavioral accuracy or reaction time in the task from which the ERN is being measured (for review see LoTemplio, Lopes, et al. 2023).

### 1.1 The Potential Role of the Simpson's Paradox

Research has failed to find a consistent and strong link between ERN amplitude and post‐error behavior (i.e., post‐error slowing, post‐error accuracy). This is likely due to a litany of possibilities, most of which are extensively discussed elsewhere (for reviews see LoTemplio, Lopes, et al. 2023; Weinberg et al. 2015) and include influences that are both biological in nature and influenced by analytical choices. However, here, we will primarily discuss the possibility that prior literature has over‐relied on inter‐individual measures of ERN amplitude and behavior (LoTemplio, Lopes, et al. 2023, table 1; Weinberg, Riesel, et al. 2012), when intra‐individual analyses are more appropriate. In other words, prior work has examined how a participant's *average* ERN amplitude correlates with their *average* post‐error slowing or post‐error accuracy (inter‐individual), rather than specifically examining how ERN amplitude on a *single trial* relates to slowing or accuracy on *the next trial* (intra‐individual). While this distinction is subtle, it is important—analyses at the inter‐individual level emphasize individual differences whereas intra‐individual analyses can isolate within‐subject trial‐to‐trial variance. If one is interested in understanding how brain activity influences behavior, the latter is typically a more direct and relevant analysis.

Importantly, even though inter‐individual analyses rely on average values derived from the trials that compose intra‐individual analyses, these two analyses do not necessarily mirror one another. Indeed, analyses at these two levels can sometimes even completely dissociate—a phenomenon more generally known as a Simpson's paradox (Kievit et al. 2013; Simpson 1951). Simpson's paradoxes are not merely theoretical—several examples demonstrate how levels of analyses can influence results, and that these discrepancies have real‐world implications. One of the most classic and notable examples is the UC Berkley admissions study (Bickel et al. 1975). In this example, the authors note that when aggregating admissions data across all departments, women were less likely to get into graduate school. However, the researchers also realized that most admissions decisions are made at the departmental level. When de‐aggregating data and examining relationships *within* departments, women were *more likely* to get into grad school—this inference was occluded in the grand average data by the fact that women tended to apply to programs that were more difficult to get into. Had the researchers only examined aggregate data, policy implications for admissions practices might have been different than what the data actually reflect.

While an interesting case study, this phenomenon is not isolated to admission data, and this phenomenon is not unique—the Simpson paradox has been documented in several areas of the psychological sciences (for review see Kievit et al. 2013), including memory research (e.g., Hintzman 1980, 1993), educational achievement (e.g., Matheson 2007), and decision‐making (e.g., Curley and Browne 2001). Perhaps one of the cases that is most relevant to the current work is an example from the literature on the P3b ERP component and response time (LoTemplio et al. 2021). In this study, we found a complete reversal of direction of the relationship between P3b amplitude and reaction time when decomposing the data from inter‐individual analyses to intra‐individual analyses. Specifically, at the subject level, participants with larger P3b amplitudes tended to slow their reaction times. However, when examining *within* subjects, participants actually responded *faster* on the trials in which they exhibited a P3b that was larger than their own average. We speculate that this reversal may reflect trait variables such as generalized response caution that may affect both response time (e.g., Ratcliff 1978) and overall P3b amplitude, as some research suggests that the P3b amplitude may reflect neural indices of evidence accumulation (e.g., Twomey et al. 2015). This is a noteworthy example because if we had only examined relationships between the P3b and RT at the subject level, we would have come to the *opposite* inference than we did about the P3b's role in facilitating responses.

Some research has suggested the Simpson's paradox is underrepresented in the literature (Pavlides and Perlman 2009), likely because people are bad at detecting it when it is present, even when prompted (Fiedler et al. 2003). Understanding whether this paradox is present is important, as one can draw completely inaccurate inferences from the data that have real‐world implications, as exemplified by the UC Berkley study. In the case of our data, we might falsely conclude that there is no relationship between ERN amplitude and post‐error behavior if we only examine at the inter‐individual level.

### 1.2 Does a Larger ERN Predict Post‐Error Behavior Adjustments?

While studies that have examined single‐trial, intra‐individual relationships between the ERN and post‐error behavior have yielded promising significant relationships (e.g., Beatty et al. 2020, 2021; Fischer et al. 2016, Kalfaoğlu et al. 2018; Cavanagh and Shackman 2015, though see Buzzell et al. 2017; Li et al. 2022; Pfabigan et al. 2021), to our knowledge, little to no work has directly compared inter and intra‐individual relationships between the ERN and *both* post‐error slowing and accuracy within the same study. Doing so would allow us to identify, using a single sample and design, whether relationships between these two levels of analyses diverge.

Beyond the risk of Simpson's paradox, the distinction between inter and intra‐individual analyses is important because choosing the wrong level of analysis could lead to inappropriate conclusions. For example, consider a researcher that is interested in whether greater ERN amplitude leads to greater post‐error slowing. The researcher then examines the relationship between subject‐average ERN amplitude and subject‐average RT on the subsequent trial and they find a positive relationship. They could conclude that ERN amplitude predicts post‐error slowing. Importantly, however, this relationship (at the level of inter‐individual variability) focuses only on between‐subject covariance (individuals with large ERNs show slowed response times on the following trials) and does not tell us information about trial‐level intra‐individual dynamics—whether a large ERN (for that participant) on a trial predicts post‐error adjustments on the next trial. This is a kind of cross‐level inference error (intimately related to the concept of ecological fallacy; Piantadosi et al. 1988) in that it attributes evidence for a particular within‐subject (trial‐level) mechanism from a between‐subject correlation. This is important to disentangle, as one could make completely incorrect assumptions about the nature of brain‐behavior relationships, and even—as our 2021 results exhibit (LoTemplio et al. 2021)—infer the complete opposite direction than is actually present in the data.

For example, it could be the case in this example that the correlation between the ERN and post‐error behavior is confounded by a trait variable such as anxiety—which has been shown to relate to both increased ERN amplitude (e.g., Hajcak et al. 2003; Meyer 2017; Weinberg et al. 2010; Weinberg, Klein, and Hajcak 2012; Xiao et al. 2011; though see Chen and Itier 2024) and post‐error behavior (e.g., van der Borght et al. 2016). Of course, this relationship could simply be present and not confounded by a third variable. However, it is difficult to infer this with this analysis technique alone. While researchers can try to control for potential trait‐level individual differences, there may be influences that researchers do not anticipate. Going back to the previous example described in Section 1.2 of the P3b and RT, we did not anticipate that trait‐level response caution was something we should potentially control for or even measure. However, this issue could be partially rectified if instead the researcher analyzed this relationship at the single‐trial level focusing on intra‐individual covariation—they could examine whether a larger ERN amplitude *for that participant compared to their own average* leads to greater post‐error slowing on the next trial. This type of analysis is agnostic to individual differences that may affect average values on both the predictor and response variable.^1^

Further, the nature of the question “does ERN amplitude predict post‐error behavior?” is fundamentally an intra‐individual question in that it implies a state‐level or trial‐by‐trial dynamic that must be tested by correlating the ERN and subsequent slowing *across trials within each participant*. This is not to say that questions about individual differences are not important; they are simply different questions.

It is also worth noting that the previous literature has focused extensively on post‐error slowing, with the underlying assumption that this slowing leads to better accuracy on subsequent trials. However, many have recently suggested that this may not inherently be the best metric of correction, as post‐error slowing may only improve accuracy under certain experimental conditions (for a review see Wessel 2018). Similarly, recent work has suggested that none of the common ways to calculate post‐error slowing are reliable (Schroder et al. 2020), and that post‐error accuracy may represent a more suitable measure of corrective behavior, as it is a more direct measure of behavioral improvements. Therefore, while the current manuscript measures both outcomes, we will especially focus on post‐error accuracy.

Finally, it is possible that the ERN does *not* predict behavior at all or that it does so in an indirect way. It is also possible that the relationships between the ERN and behavior align at both levels of analysis. Yet, in order to more confidently conclude this, it would be useful for a project to directly examine inter and intra‐individual relationships to post‐error behavior adjustments. To this end, understanding relationships between *other* physiological error‐processing responses and post‐error behavior may be useful.

### 1.3 Does the Pe Predict Post‐Error Behavior Adjustments?

Beyond the ERN, there are other relevant physiological signals that index error‐processing. One of which is the positivity following an error (Pe). The Pe is a positive deflection in the waveform that occurs after the commission of an error and directly follows the ERN, approximately 200–500 ms following an error (Overbeek et al. 2005; Falkenstein et al. 1991). Much less is known about the Pe than the ERN, but it is thought to represent an individual's conscious awareness of having made an error whereas the ERN is thought to represent merely error detection (e.g., Di Gregorio et al. 2018; Overbeek et al. 2005), suggesting separable processes. Thus, it is possible that the Pe might predict separate variance in behavior. For example, it is possible that conscious awareness of an error, as indexed by Pe amplitude, may generate greater behavior adjustment *due* to the conscious awareness.

Further, some research suggests that the Pe is a marker of evidence accumulation in the brain to mark the recognition of an error (Steinhauser and Yeung 2010). The authors demonstrated that the Pe was larger when this stronger evidence was required to recognize an error. Though the authors did not examine relationships between Pe amplitude and behavior on the subsequent trial, the current work does demonstrate that the Pe does appear to reflect evidence accumulation for conscious recognition of an error. This recognition of an error is likely to lead to adjusted behavior (Steinhauser and Yeung 2010).

Fewer studies have examined the relationship between the Pe and post‐error behavior, and most have examined relationships between subject‐average Pe amplitude and average PES. For example, a few studies found that increased average Pe amplitude led to increased average PES (e.g., Chang et al. 2014; Nieuwenhuis et al. 2001; Hajcak et al. 2003). Yet, at least one other study documented no relationship between average Pe amplitude and average PES, but that there was a relationship between average Pe amplitude and average post‐error accuracy (Schroder et al. 2012). In their review of post‐error slowing metrics, Schroder et al. (2020) articulate that the limited research on the Pe and post‐error behavior has also been dominated by a focus on PES rather than post‐error accuracy. Additionally, we are unaware of significant work comparing subject‐average relationships of Pe amplitude and PEA to single‐trial relationships of these variables.

### 1.4 Does Error‐Related Pupil Dilation Response (PDR) Predict Post‐Error Behavior Adjustments?

Finally, previous research using pupillometry has also established that when individuals make mistakes, a large pupillary dilation is observed compared to correct responses (Braem et al. 2015; Critchley et al. 2005; LoTemplio, Silcox, et al. 2023; Maier et al. 2019; Murphy et al. 2016; Rondeel et al. 2015; Wessel et al. 2011), called the error‐related PDR. The exact function of the error‐related PDR is also currently under debate. However, some suggest that this response may reflect an orienting response to unexpected motor errors. Supporting this, Braem et al. (2015) found that PDRs to errors were larger in an easier task when they were less expected. If the PDR does represent an orienting response to errors, there are multiple paths by which this could influence behavior. On the one hand, this orienting response may be disruptive and cause a delay in responses on following trials, or perhaps even mistakes. On the other hand, if the inter‐stimulus‐interval or response–stimulus‐interval is long, and the orienting response resolves, it may be adaptive to the individual and help them to avoid future mistakes (for reviews see Wessel 2018; Murphy et al. 2016). Given this, we should expect that either way there should be a relationship between PDR amplitude and behavior—whether adaptive or maladaptive.

Few prior experiments have studied the error‐related PDR at all, and even fewer have reported whether it predicts post‐error accuracy or RT. Murphy et al. (2016) found that larger PDR amplitude did indeed predict both greater post‐error slowing and a higher likelihood of accuracy on the next trial. However, this study used an unusually long inter‐stimulus‐interval (ISI) of 6000 ms, or 6 s, and a 4‐choice reaction time task. Therefore, if the error‐related PDR reflects the orienting response, it is unclear if its amplitude would correspond to next‐trial behavior at shorter ISIs or in a canonical 2‐choice reaction time task. Maier et al. (2019) recorded pupillary data while subjects performed a Flanker task with a variable response–stimulus interval that was randomly drawn from 1500 to 2500 ms. They also found that the amplitude of the PDR predicted post‐error behavior accuracy, but only for flanker errors (errors associated with the distracting stimulus) and not non‐flanker errors (errors not associated with either the target or distracting stimulus).

Though these two studies demonstrate preliminary evidence of a link between PDR amplitude and post‐error behavior adjustment, given the inconsistencies linking other physiological error responses to behavior, replication and extension of this work would be valuable. Further, it is unclear the extent to which the PDR predicts variance above and beyond ERN or Pe amplitude.

### 1.5 Relationships Among the ERN, the Pe, and the Error‐Related PDR

Finally, it is important to understand relationships among these three error‐sensitive measures, as they may reflect either similar underlying neural circuits and processing streams or distinct aspects of error‐processing. This also has important implications for studying how psychophysiological indices predict behavior adjustment. For example, it could be possible that one psychophysiological index could represent an orienting response that caused *slowing* after an error, but another reflects recruitment of control processes, which could cause *accuracy* adjustments. As an initial step, we need to first understand the extent to which these three indices covary. Further understanding of the relationships among these measures may also be useful when making decisions about inclusion of error‐processing indices in studies.

Much of the prior experimental work has been dominated by separate univariate analyses examining whether manipulated variables had similar patterns of effects on the ERN and the Pe, and sometimes the PDR. Prior work has shown that both the Pe and the error‐related PDR were sensitive to working memory load, while the ERN was not (LoTemplio, Silcox, et al. 2023). Yet, little is known about how the two signals relate to one another. The need for examining such correlations among outcomes has been established in other literatures. For example, in the oddball paradigm, rare stimuli evoke both a large P3b and a large PDR, but these effects have been consistently uncorrelated with each other (LoTemplio et al. 2021; Kamp and Donchin 2015; Murphy et al. 2011), providing important insight suggesting that though similar conditions may elicit both components, they may be functionally and neurologically distinct processes—an inference that is easy to miss without directly examining relationships among variables.

Furthermore, understanding these relationships can generate insight into potential underlying mechanisms tied to specific components. For example, Rommers et al. (2017; figure 5) examined the relationship between ERP components and activity in the time‐frequency domain that occurred during the same time period. They observed a correlation between language‐related ERP components and mid‐frontal theta, which has been consistently linked to cognitive control processes (e.g., Cavanagh et al. 2012; Cavanagh and Frank 2014). These findings collectively suggested that language‐related late positive components were related to the engagement of cognitive control processes during language processing (Rommers et al. 2017).

Some have suggested that the Pe and PDR share similar neurophysiological underpinnings (Ridderinkhof et al. 2009). The cognitive PDR response reflects, among other things, phasic locus coeruleus norepinephrine (LC‐NE) activity (for review see Joshi and Gold 2020). Comparatively fewer studies have examined norepinephrine contributions to the Pe. On the one hand, studies have suggested a link between the Pe and the attentional orienting response (for review see Wessel 2018), which is thought to be at least partially driven by LC‐NE activity that also drives increases in PDR amplitude (e.g., Gabay et al. 2011). On the other hand, one study found that oral administration of Yohimbine, which increases norepinephrine release, did not affect Pe amplitude (Riba et al. 2005), suggesting separability of the Pe and PDR. However, to our knowledge, direct comparison of the Pe and PDR has not been made.

Similarly, despite multiple studies examining the ERN and PDR simultaneously, few, if any, have examined how the two correlate with one another. It is also possible that the ERN and PDR both share underlying norepinephrine influences. For example, while Yohimbine did not influence Pe amplitude, the authors found that it *did* influence ERN amplitude (Riba et al. 2005). An examination of the correlations among these measures would further our understanding into their convergence and divergence (for review see Clayson 2024), yielding potential insight into the degree of separability of underlying processes.

Finally, there has historically been debate as to the degree to which the ERN and Pe represent separable processes (Overbeek et al. 2005). Current research seems to suggest that the Pe represents a process of error monitoring that is distinct from the ERN, and that can occur even when an ERN does not (e.g., Di Gregorio et al. 2018; Charles et al. 2013; Pfister et al. 2016; Woodman 2010). For example, one study found that a Pe was present even when participants did not have access to a “correct” representation but knew that they made an error, whereas the ERN was not present (e.g., Di Gregorio et al. 2018), further supporting the theory that the Pe codes for error awareness processes, whereas the ERN may code for mismatch between the stored correct response against the incorrect actual response. Furthermore, Clayson and colleagues recently examined divergent and convergent validity among the ERN, Pe, and other control‐related components at the between‐subjects level (Clayson, Mcdonald, et al. 2025). They found evidence for divergent validity of the ERN and Pe (both on incorrect trials and the ERP difference scores), further suggesting differentiation of the two components (see also Riesel et al. 2013).

Yet, to our knowledge, no work has directly compared relationships between the ERN and Pe at both the inter‐individual and intra‐individual (single‐trial) level. As described earlier, these relationships can completely dissociate depending on what level of analysis you select, and one level of analysis may be more appropriate than another depending on the research question. Thus, replication and extension of the observed relationships in Clayson, Mcdonald, et al. (2025) may be useful to better understand relationships between these components on a trial‐by‐trial basis to avoid potential subject‐level confounds.

### 1.6 Summary and Current Study

Given the nebulous understanding of how various physiological measures of error‐processing relate to task behavior (and to one another), we report a secondary data analysis of previously published research (LoTemplio, Silcox, et al. 2023) to further elucidate relationships among these outcomes. We aim to answer the following research questions, with the following hypotheses:

1. Does within‐person single‐trial variability in the ERN, Pe, or PDR predict slowing or accuracy on the next trial? If so, does this effect differ from subject‐level average correlations?

We hypothesize that there will be a weak but significant relationship between single‐trial ERN amplitude and post‐error slowing such that as ERN amplitude increases, slowing increases. We hypothesize that there will be no relationship between subject‐level ERN and post‐error slowing. Given our hypothesis that the PDR and Pe might be more strongly related to task behavior, we hypothesize that the Pe and PDR will similarly show a stronger relationship to post‐error slowing at the single‐trial level compared to the ERN. As with the ERN, we also hypothesize that there will be no relationship between subject‐level Pe and PDR and subject‐level post‐error slowing.

We hypothesize that there will be a weak but significant relationship between single‐trial ERN and post‐error accuracy, such that as ERN amplitude increases, accuracy increases. We hypothesize that there will be no relationship between subject‐level ERN and post‐error accuracy. Given prior works' suggestions that the PDR and Pe might be more strongly related to task behavior, we hypothesize that the Pe and PDR will similarly show the same relationship to post‐accuracy behavior as the ERN, but to a larger effect. We hypothesize that there will be no relationship between subject‐level Pe and PDR and subject‐level post‐error accuracy.

2 Does the single trial amplitude of the Pe or ERN predict pupillary amplitude? Does this differ from subject‐level average amplitude?

As some suggest that the Pe and PDR share neural overlapping origins, we expect that single trial Pe amplitude should predict PDR amplitude, such that as Pe amplitude increases, so does PDR amplitude. As prior work suggests that the ERN initiates a series of downstream processes that may adjust behavior, and some of these processes might include the Pe or PDR, we predict that the ERN will predict PDR amplitude, but to a lesser extent than Pe amplitude. We predict that we will only observe these relationships at the single‐trial level. While less of the goal of the current manuscript, we also examine relationships between the ERN and the Pe at both the intra‐individual and inter‐individual levels. While prior work has suggested that these two are separable components (e.g., Clayson, Mcdonald, et al. 2025), we hope that analysis may be helpful in further clarifying the relationship (or lack thereof) between the ERN and Pe at multiple levels of analysis. We predict no significant relationship between the ERN and Pe amplitude at either level of analysis.

## 2 Method

### 2.1 Sample Size Planning and Participants

We recruited 73 participants to complete the study (LoTemplio, Silcox, et al. 2023). However, several participants were excluded for failing to make at least 7 errors (Meyer et al. 2013; *n* = 13), or due to equipment issues during recording (*n* = 7). This resulted in a final sample of 53 participants. The self‐identified gender break‐down of these participants was 33 women, 2 nonbinary, and 18 men. The self‐identified racial break‐down included 44 White participants, 2 Asian participants, 4 Latinx participants, and 3 multi‐racial participants. Participants ranged in age from 18 to 34 (mean age = 25.48; SE = 0.62). Participants reported no major neurological disorders (i.e., seizure disorders). Two participants self‐disclosed ADHD diagnoses, and no participants reported any generalized anxiety or major depressive disorders. All participants had normal or corrected‐to‐normal vision.

### 2.2 Procedure

The protocol was approved by the University of Utah's IRB. The study was completed in a single session. Upon arrival to the study, participants were first given a verbal explanation of the study and then given a written consent document also explaining the study and asking participants if they consented to participate. After providing written consent to participate in the study, participants first completed the 6‐item State–Trait Anxiety Inventory (STAI; Spielberger 2010; Tluczek et al. 2009) to assess state anxiety and the Penn State Worry Questionnaire (PSWQ; Meyer et al. 1990) and the Brief Worry Questionnaire (Verkuil et al. 2021) to measure trait anxiety. These questionnaires were included because anxiety is known to relate to ERN amplitude (Hajcak et al. 2003; Meyer 2017; Moser et al. 2013; Weinberg et al. 2010; though see Chen and Itier 2024). Overall, our trait anxiety scores on the PSWQ were approximately normally distributed and the mean score of our sample was 54.25 (SE = 1.75) out of a range of 16–80 (higher score = higher trait worry) indicating that we didn't obtain a particularly high or low trait‐worry sample. After completing their anxiety questionnaires, participants were fitted with both an EEG cap and set up with a desktop‐mounted eye tracker for pupillometry. Participants then completed a series of 24 blocks of 50 trials an Ericksen flanker task. In this task, both anxiety and working memory load were manipulated in a 2 × 2 within‐subjects design. However, given that we observed no differences in ERN amplitude according to state anxiety manipulation (LoTemplio, Silcox, et al. 2023), a finding that was recently replicated in another study (Härpfer et al. 2022), we decided to collapse across high and low anxiety conditions in the current study. We also collapsed across both working memory load conditions (high load vs. low load).^2^ By collapsing four experimental conditions or cells, we were able to increase the number of error trials included in the models, maximizing statistical power. For more information about the experimental manipulations, refer to LoTemplio, Silcox, et al. (2023). Importantly, there was an equal number of high versus low anxiety trials and high versus low working memory trials, so collapsing across conditions is also likely to wash out differential effects.

#### 2.2.1 Flanker Task and Behavioral Data

Participants completed 24 blocks of a flanker task (50 trials each) for a total of 1200 trials. For each block, the Flanker task consisted of a high‐congruency (80% congruent, 20% incongruent) version of the Eriksen and Eriksen (1974) Flanker Task created in PsychoPy (3.2.0). Participants were instructed to respond based on the identity of the centrally presented letter in a series of five‐letter horizontal arrays. There were two types of stimuli: congruent and incongruent. A congruent stimulus consists of all identical letters (e.g., SSSSS or HHHHH), and an incongruent stimulus consists of “flanking” letters that are associated with the opposite response (e.g., SSHSS or HHSHH). Each stimulus was preceded by a fixation cross, presented for 1400 milliseconds (ms) in the center of the display, followed by a blank screen for 100 ms, for a total of 1500 ms response–stimulus interval (RSI). Stimuli remained in view until the participant responded. On each trial, we measured participants' reaction times (ms) and accuracy using a MilliKey Button Box (Halifax, Canada). We also measured reaction time on the next trial (post‐error slowing; PES) using the robust PES metric (Dutilh et al. 2012), and accuracy on the following trial (post‐error accuracy; PEA). For robust PES for each participant, we windsorized extreme outliers (the bottom and top 0.25% of data) that were infeasibly fast or due to a distraction that caused an extreme delay in response.

#### 2.2.2 EEG and Pupillometry Recording

We capped participants with actiCap 32 active electrode channel gel‐based cap in accordance with the 10–20 system (Jasper 1958). We also placed an electrode on the left infraorbital ridge to monitor for vertical eye movements and blinks, and another two electrodes on the outer canthus of each eye to monitor for horizontal eye movements. Electrode impedances were kept below 5 kΩ. EEG was acquired from the BrainAmp DC (BrainVision LLC). EEG data were continuously recorded in DC mode with a low‐pass filter of 1000 Hz (fifth‐orderButterworth filter with a roll‐off of 30 dB/octave). We amplified the continuous EEG waveform with a BrainAmp DC (Brain‐Vision LLC, Morrisville, NC) amplifier at a sampling rate of 500 Hz. We instructed participants to maintain their fixations on the fixation cross and stimuli and to limit blinks and movements as much as possible.

Pupil data were recorded with an Eye‐Link 1000 desktop‐mounted Eye Tracker at a sampling rate of 1000 Hz. The eye tracker viewing was binocular, but we only recorded pupil dilation from the right eye. We placed participants' heads in a chinrest to aid in minimizing their head movement. A fixation correction was presented between each block to check that the system was still correctly calibrated. In cases where a minor drift occurs, the system checks before the beginning of the trial. If the calibration was lost, the experiment was paused, and the eye‐tracking system was re‐adjusted.

### 2.3 EEG Processing and Analysis

We downsampled data to 250 Hz and bandpass filtered from 0.1 to 30 Hz with a Butterworth filter type and a 12 dB/octave roll‐off. We epoched the data from −400 to 400 ms relative to response onset, with a −400 to −200 ms baseline period. We sorted trials into correct vs. incorrect trial bins. Eye movements were corrected using Gratton's regression method of eye movement correction (Gratton et al. 1983). To ensure that the algorithm missed no blinks, we then ran the data through a moving‐window artifact rejection function. An epoch was rejected entirely if the VEOG channel's data deflects more than 100 microvolts within a 20 ms time period. If a trial was rejected in the pupillometry recording (described below), it was also rejected in the EEG data, and vice versa. Participants' data were removed if fewer than 7 error trials survived artifact correction (Meyer et al. 2013). The mean number of correct trials included for each participant was 962.01 (SE = 17.19, range = 605–1131) and the mean number of error trials was 60.84 (SE = 5.53; range = 12–198). We then subtracted the baseline period from each epoch, and extracted each accepted epoch individually from MATLAB.

To select our time window, we used a collapsed localizer approach (Luck and Gaspelin 2017). To accomplish this, we used the aggregate waveform of all subject averages in all conditions to identify the peak latency of the overall ERP difference wave (error‐correct). We chose a priori to define a window of ±25 ms around the peak. For this sample, the average peak latency was 40.18 ms (SE = 2.62). Therefore, we use the measurement window of 15–65 ms to measure the mean amplitude of our ERN effects for each subject and for each trial. Our collapsed localizer also demonstrated that FCz produced the largest error effect across all subjects. Therefore, we used FCz as the electrode from which to calculate mean amplitude from each subject and amplitude at each trial. For the Pe, we used the same collapsed localizer technique, with an a priori window size of 200 ms, which was ±100 ms around the peak latency (see Overbeek et al. 2005). We identified a peak latency of 241 ms and that the component was maximal at electrode Pz. Therefore, we examined the amplitude of this component at electrode Pe using a window from 141 to 341 ms. Note that these window sizes and electrode selections also mirror the analyses in the previous study using the same data (LoTemplio, Silcox, et al. 2023).

Finally, we measured ERP amplitude at both the single trial and subject level. To measure single trial ERN amplitude, we took the mean amplitude from 15 to 65 ms for each error and correct trial. We then subject‐mean centered all trials to each subject's mean of all trials. We performed this centering technique in order to isolate within‐subjects variance (Enders and Tofighi 2007), allowing us to avoid the “uninterpretable blend” (Raudenbush 2002, 139) of the inclusion of both within‐subjects and between‐subjects variance to the estimate of the fixed effect. To assess subject‐level mean amplitude for the ERN, we took the mean of each subject's correct trials from 15 to 65 ms and the mean of each subject's incorrect trials from 15 to 65 ms. We grand‐mean centered these subject‐average variables to aid in interpretation. We repeated the same process for the Pe, but with the measurement window 141–341 post response, and at electrode Pz. We also subject‐mean centered the Pe predictor variable at the intra‐individual level, and grand‐mean centered at the inter‐individual level.

### 2.4 Pupillometry Processing and Analysis

All pupil data were processed using the R language for statistical computing (version 4.5.1; R Core Team 2022) and closely aligned with best practices outlined by Kret and Sjak‐Shie (2019) and previously successfully used by our group across multiple studies (LoTemplio et al. 2021, LoTemplio, Lopes, et al. 2023; LoTemplio, Silcox, et al. 2023, 2024; Silcox and Payne 2021). The pupil data were first epoched from −200 to 1100 ms around participants' responses using the EyeLink DataViewer software. The epoched pupil data were then imported into R and processed in four steps in the same procedure as in LoTemplio, Silcox, et al. (2023). First, we removed any dilation speed outliers, as our eye tracker will occasionally temporarily identify something as part of the pupil that is not (i.e., eyelash, etc.). The second step was to identify and remove eye blinks. When participants blink, the eye tracker temporarily loses track of the pupil. Therefore, we identified eye blinks via gaps in the data. Eye blinks were identified as gaps in the data of at least 80 ms. Once the eye blinks were identified, 50 ms of data were removed on either side of the blink. If more than 50% of the data on a trial was missing after these first two steps, the trial was rejected from further processing and analysis. The third step was to use linear interpolation to fill in gaps in the data. The fourth step was to filter the data using a low‐pass 12 dB/octave Butterworth filter with a half‐amplitude cut‐off of 10 Hz.

In the SR‐Eyelink 1000 eye tracker, pupil size is measured in arbitrary units which are based on the number of thresholded pixels that make up the pupil, but these units have been shown to map directly onto the physical pupil diameter (Hayes and Petrov 2016). To measure the PDR, we measure change in these arbitrary units that occur after the participant makes a response, as a percent change from a given pre‐response baseline period. We examined the phasic PDR to errors from −200 to 1100 ms, which was the largest window we possibly could examine given experimental constraints. We baselined from −200 to −100 ms pre‐stimulus to avoid any potential error‐related preparatory activity between −100 to 0 that could skew results.

Next, to identify the measurement window for the error effect of the pupillometry response, we used the same window (180–1100 ms post response) as in our prior study (LoTemplio, Silcox, et al. 2023) for every individual trial to calculate single trial PDR amplitude. This window was originally defined by using a mass univariate technique (e.g., LoTemplio, Silcox, et al. 2023) on the average collapsed difference wave of error‐correct pupillary responses. This difference wave was calculated for each subject by averaging all error trials that a subject made and all correct trials, and then subtracting their average correct waveform from their average error waveform. Using these difference waveforms, we computed mass univariate testing (Groppe et al. 2011) by running a one‐sample *t*‐test at each time point, looking for time windows in which the data were significantly different from zero. We corrected the *t*‐tests using false discovery rate correction (Benjamini and Hochberg 1997). This methodology returned a time window of 180–1100 ms post‐response. This time window was used to calculate the mean PDR amplitude for each subject as a percentage change from the pre‐response baseline period. We then calculated each subject's mean pupil dilation response (PDR) using difference waves (error‐correct), for use in our linear mixed‐effects models (see below). We also used this window for each individual trial to determine single‐trial amplitude. For intra‐individual PDR amplitude predictor variables, we subject‐mean centered, as described above, and for inter‐individual PDR predictor variables, we grant‐mean centered, as described above.

### 2.5 Analytical Plan

The analytical plan can be grouped into roughly two portions: (1) Relationships between physiological indices of error‐processing and post‐error slowing and post‐error accuracy, and (2) Relationships between PDR and ERP responses.

#### 2.5.1 Relationships Between Physiological Indices of Error‐Processing and Post‐Error Slowing

To address the *first set of hypotheses* to understand the relationship between physiological indices of error‐processing and post‐error slowing, we conducted linear mixed‐effects models using R's lme4 package (Bates et al. 2015), using subject as a random intercept to control for non‐independence of observations. Fixed‐effects predictors included subject‐mean centered ERP or PDR amplitude. Only error trials were included. We used R's *afex* package (v1.0‐1, Singmann et al. 2015) to conduct likelihood ratio tests on this model to assess the main effects. We used the *lmertest* package to generate beta values.

For each model, the outcome variable was robust PES, while the fixed‐effect physiological predictors were (1) single‐trial ERN amplitude, (2) single‐trial Pe amplitude, and (3) single‐trial PDR amplitude, respectively. All single‐trial physiological predictors were subject‐mean centered in each model. Only error trials were included in the models, as including both correct and error trials resulted in a singular fit of the model, and the primary interest was in post‐*error* slowing.

For each of these models, we also added the fixed effect to the model as a random slope to test, using likelihood ratio tests, whether the model fit is improved compared to a model with no random slopes (e.g., Meteyard and Davies 2020). This enabled us to ensure that we were following principles to “keep it maximal” with random effects (i.e., Barr et al. 2013). For each of the models, none of the additions of single‐trial amplitude as random slopes improved the models, and so they were not added. Final model selections are also visible in Table 3.

For symmetry with the above model, at the subject level, we also only examined relationships between the physiological outcome variables on error trials to post‐error reaction time. For these models, a general linear regression was run. For each model, the outcome variable was subject‐average robust PES, and the predictors were (1) subject‐level ERN amplitude, (2) subject‐level Pe amplitude, and (3) subject‐level PDR amplitude. Models and all included terms are visible in Table 4.

#### 2.5.2 Relationships Between Physiological Indices of Error‐Processing and Post‐Error Accuracy

Next, we ran models to understand how our single‐trial physiological variables on error trials influenced behavioral accuracy on the next trial. In other words, if the amplitude of the ERN, Pe, or PDR was larger on a given trial, was that associated with an increased chance of a correct response on the next trial? Again, for these models, we only used error trials.

To answer this research question, we conducted a generalized logistic regression using *glmer()* in R's *lme4* package. We used the same model and random effects structure selection technique as above. We ran three models. Each included a subject‐mean centered single trial physiological variable as a fixed‐effects predictor (ERN, Pe, or PDR), and next‐trial accuracy (correct vs. incorrect) as the outcome variable. The regression equation output log‐odds, which we transformed into an odds ratio. We then used the intercept and the odds ratio to calculate and plot the model‐estimated chances of post‐error accuracy given the ERN, Pe, and PDR amplitude on a given trial. For the Pe and ERN models, the inclusion of single‐trial amplitude as a random slope did not improve fit, so they were not included. However, the addition of PDR amplitude as a random slope across subjects improved model fit (*χ* ^2^(2) = 7.15, *p* = 0.03), so it was included. Final model selections are also visible in Table 3.

For subject‐level analyses, a general linear regression was run. For each model, the outcome variable was subject‐average PEA, and the predictors were (1) subject‐level ERN amplitude, (2) single‐trial Pe amplitude, and (3) single‐trial PDR amplitude. Models and all included terms are visible in Table 6.

#### 2.5.3 Relationships Between the ERPs (Pe and ERN) and PDR Response

To assess the relationships between the ERPs and the PDR responses, we used the same model structure and random effects selection technique as for post‐error behavior adjustment. However, in this set of models, fixed‐effects predictors included not only subject‐mean centered ERP amplitude, but also trial accuracy (error trial vs. correct trial), and the interaction between the two terms. To facilitate the interpretation of significant interactions and pairwise comparisons, we used the *pairs*() function from the *emmeans* package in R (v1.4.3.01, Lenth et al. 2018).

The first model included single‐trial ERN amplitude (subject‐mean centered) as a fixed‐effects predictor of single trial PDR amplitude. The second model was identical, except it included Pe amplitude (subject‐mean centered) as the fixed‐effects predictor of single trial PDR amplitude. The third was also identical, except it included ERN amplitude (subject‐mean centered) to predict Pe amplitude. For all three models, we added the fixed effects of trial accuracy (error trial vs. correct trial) and the interaction between either the ERN or Pe and accuracy. For all three single trial models, a random slope of accuracy improved the model fit, so it was added. All terms in the final model selections are also visible in Table 1.

**TABLE 1:** Models for examining intra‐individual relationships between ERN, Pe, and PDR and robust post‐error slowing (PES), *N* = 53 subjects, 3199 observations (errors only).

Predictors	Beta Estimates	SE	χ^2^	df	*p*
Model 1: ERN on PES					
(Intercept)	31.53	3.92	—	—	—
ERN amplitude	0.39	0.27	1.94	1	0.16
*Random effects*	*σ* ^2^	*τ*_00 (Subject)_	*τ*_11_	*ρ*_01_	
	27,198.05	147.93	—	—	
Model 2: Pe on PES					
(Intercept)	22.99	4.03	—	—	—
Pe amplitude	0.81	0.29	8.11	1	< 0.01*
*Random effects*	*σ* ^*2*^	*τ*_00 (Subject)_	*τ*_11_	*ρ*_01_	
	27,154.20	135.70	—	—	
Model 3: PDR on PES					
(Intercept)	27.94	3.92	—	—	—
PDR amplitude	21.64	37.36	0.33	1	0.57
*Random effects*	*σ* ^2^	*τ*_00 (Subject)_	*τ*_11_	*ρ*_01_	
	27,217.88	139.39	—	—	

*= *p* < .05.

We also examined the relationships between the ERN and the PDR, the Pe and the PDR, and the ERN and the Pe at the subject‐level. For these models, we calculated the average amplitude of each component for correct and incorrect trials. A linear mixed‐effects model with subject as a random intercept was run with subject‐level ERN as a fixed effect, as well as accuracy, and the interaction between the two, with average PDR amplitude as the outcome variable. A second mixed‐effects model was run identical to the above except with the Pe as a predictor, and a third was identical but included average ERN amplitude as the predictor to average Pe amplitude as the outcome. The same model selection technique used for single trial data (described above) was also used. In the results section, we detail what the final model selection was for each research question—no random slopes improved model fit for these models. All terms in the final model selections are also visible in Table 2.

**TABLE 2:** Models for examining inter‐individual relationships between ERN, Pe, and PDR and robust post‐error slowing (PES), *N* = 53 subjects, averages computed on error trials only.

Predictors	Beta estimates	SE	*t*	df	*p*
Model 1: ERN on PES					
(Intercept)	31.10	4.34	—	—	—
ERN amplitude	−3.01	1.05	−2.87	51	< 0.01*
Model 2: Pe on PES					
(Intercept)	31.10	4.65	—	—	—
Pe amplitude	0.89	1.10	0.81	51	0.420
Model 3: PDR on PES					
(Intercept)	31.10	4.67	—	—	—
PDR amplitude	−10.12	124.50	−0.08	51	0.94

*= *p* < .05.

## 3 Results

### 3.1 Inter and Intra‐Individual Relationships Between Physiological Variables of Error‐Processing and Post‐Error Slowing

**FIGURE 1:**
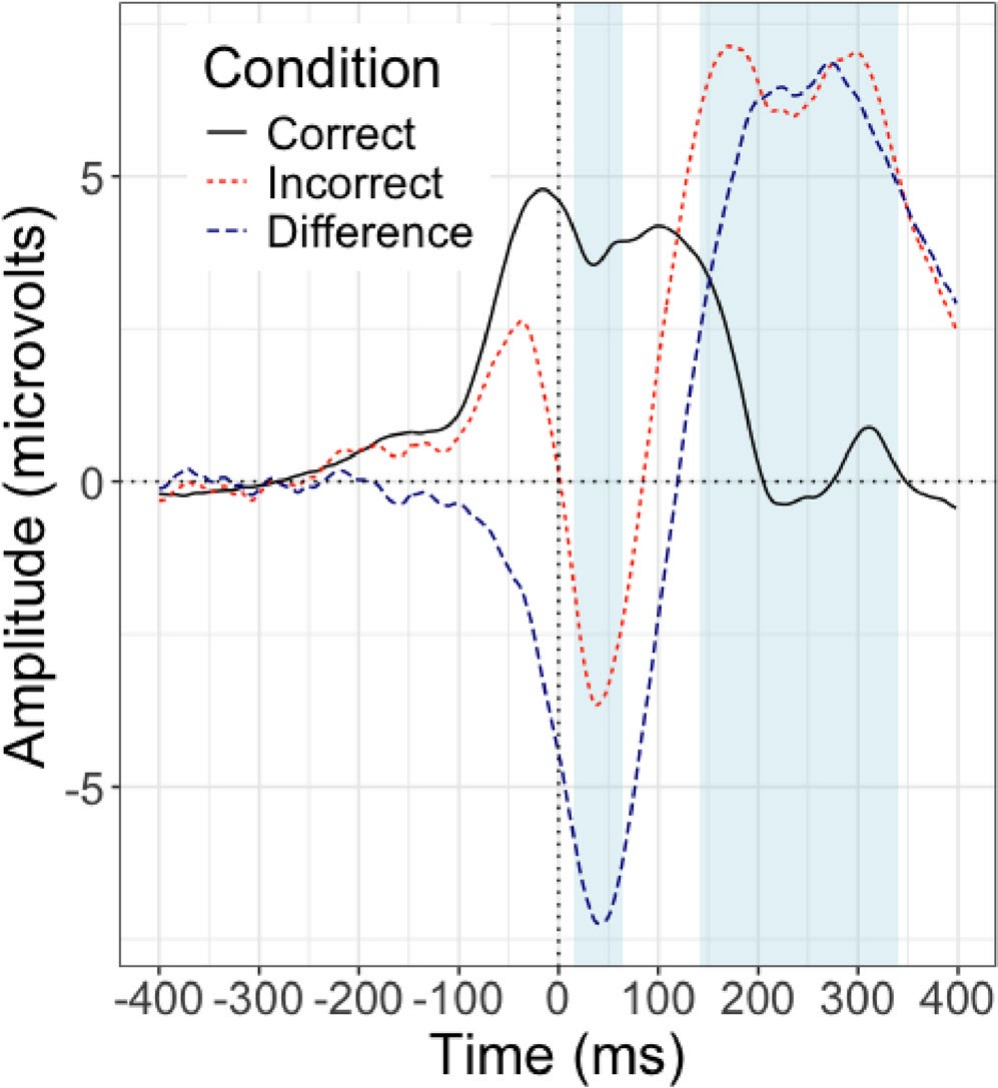
ERP amplitude in response to errors (red), correct responses (black), and the difference wave between the two (dark blue). Response at time 0 ms. Shaded portions represent the measurement window for the ERN (15–65 ms) and the Pe (141–341 ms).

**FIGURE 2:**
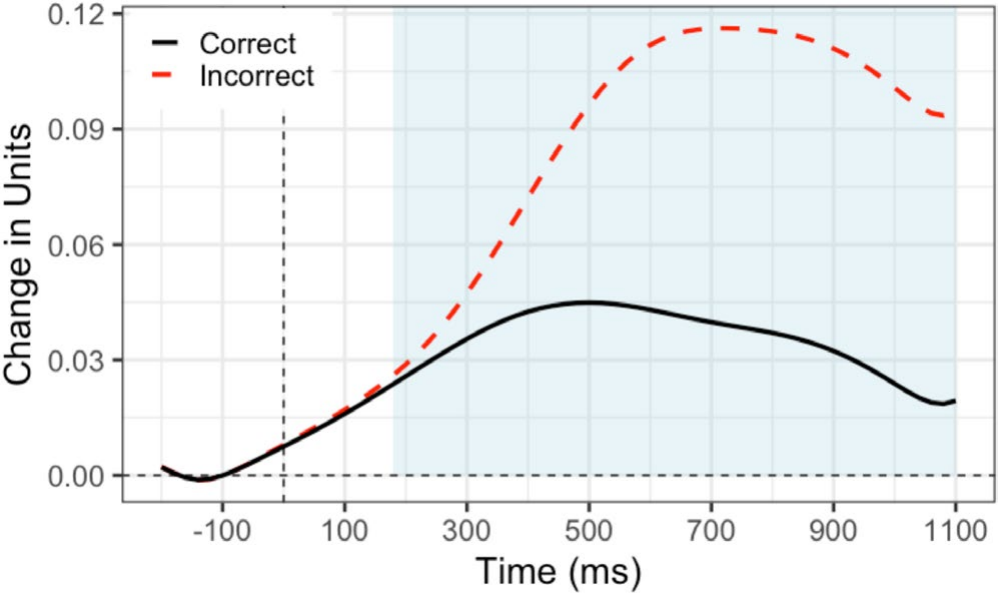
Pupillary amplitude in response to incorrect and correct responses. Response at time 0 ms. Shading represents measurement window (180–1100 ms).

**FIGURE 3:**
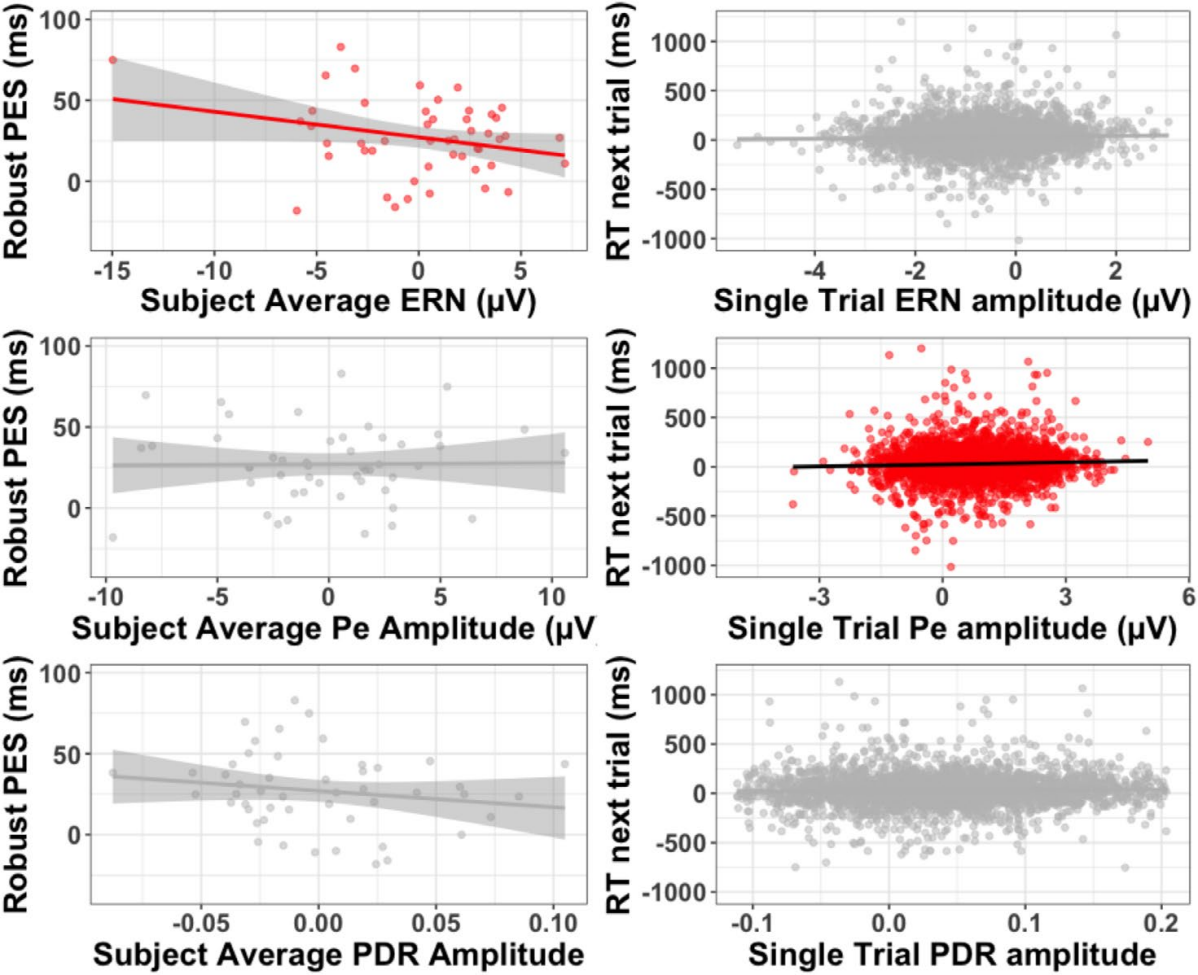
The left panel depicts subject‐level relationships between average ERN, Pe, and pupillary amplitude and average robust post‐error slowing for error trials only. Lines of best fit are drawn. Right panel depicts single‐trial relationships between subject‐mean centered Pe, ERN and pupillary amplitude and single trial robust PES (for errors only). Lines of best fit are drawn. For all subplots, red colors lines indicate a significant relationship. Y‐axes are set to ±2 SD of the *y* variable (Witt 2019).

General error effects for EEG and pupillometry can be viewed in Figures 1 and 2 respectively. First, we explored both inter and intra‐individual relationships between our physiological error‐processing variables and post‐error slowing. Recall that for both model types (inter and intra‐individual), we only included error trials to examine post‐error slowing. Results of all models are visualized in Figure 3. It is worth noting that the average reaction time for each participant was 474.48 ms (SE = 14.69), and the average accuracy was 94.11% (SE = 0.51%), consistent with prior work using this version of the flanker task (e.g., Coleman et al. 2018; LoTemplio et al. 2020).

The addition of ERN amplitude as a random slope across subjects did not improve model fit (*p* > 0.05), so it was not included. We found no effect of single‐trial ERN amplitude on post‐error slowing (See Table 1; Figure 3). For subject‐level ERN, we did find a significant effect of average ERN amplitude to average robust PES (See Table 2; Figure 3), such that individuals with a more negative (larger) ERN amplitude showed greater post‐error slowing on the following trial.

Next, we examined how Pe amplitude affected PES, using the same model structure as above. The inclusion of Pe amplitude as a random slope did not improve model fit (*p* > 0.05), so it was not included. We found that intra‐individual variation in Pe amplitude significantly predicted post‐error slowing (See Table 1; Figure 3), such that trials with a larger Pe amplitude within subject were associated with greater slowing on the following trial. We found no significant relationship between subject‐average Pe amplitude and subject‐average robust PES (See Table 2; Figure 3).

Finally, we examined how variation in PDR amplitude affected PES. The inclusion of PDR amplitude as a random slope across subjects did not improve model fit (*p* > 0.05), so it was not included. We found no relationship between single‐trial PDR amplitude and post‐error reaction time (See Table 1; Figure 3). We also found no significant effect of subject‐average PDR amplitude on subject‐average robust PES (See Table 2; Figure 3). Therefore, PDR amplitude did not predict post‐error slowing at either the intra or inter‐individual level. In summary, the ERN was the only predictor to significantly predict PES at the subject level, while the Pe was the only predictor to predict slowing on the next trial at the single‐trial level.

### 3.2 Inter and Intra‐Individual Relationships Between Physiological Variables of Error‐Processing and Post‐Error Accuracy

**FIGURE 4:**
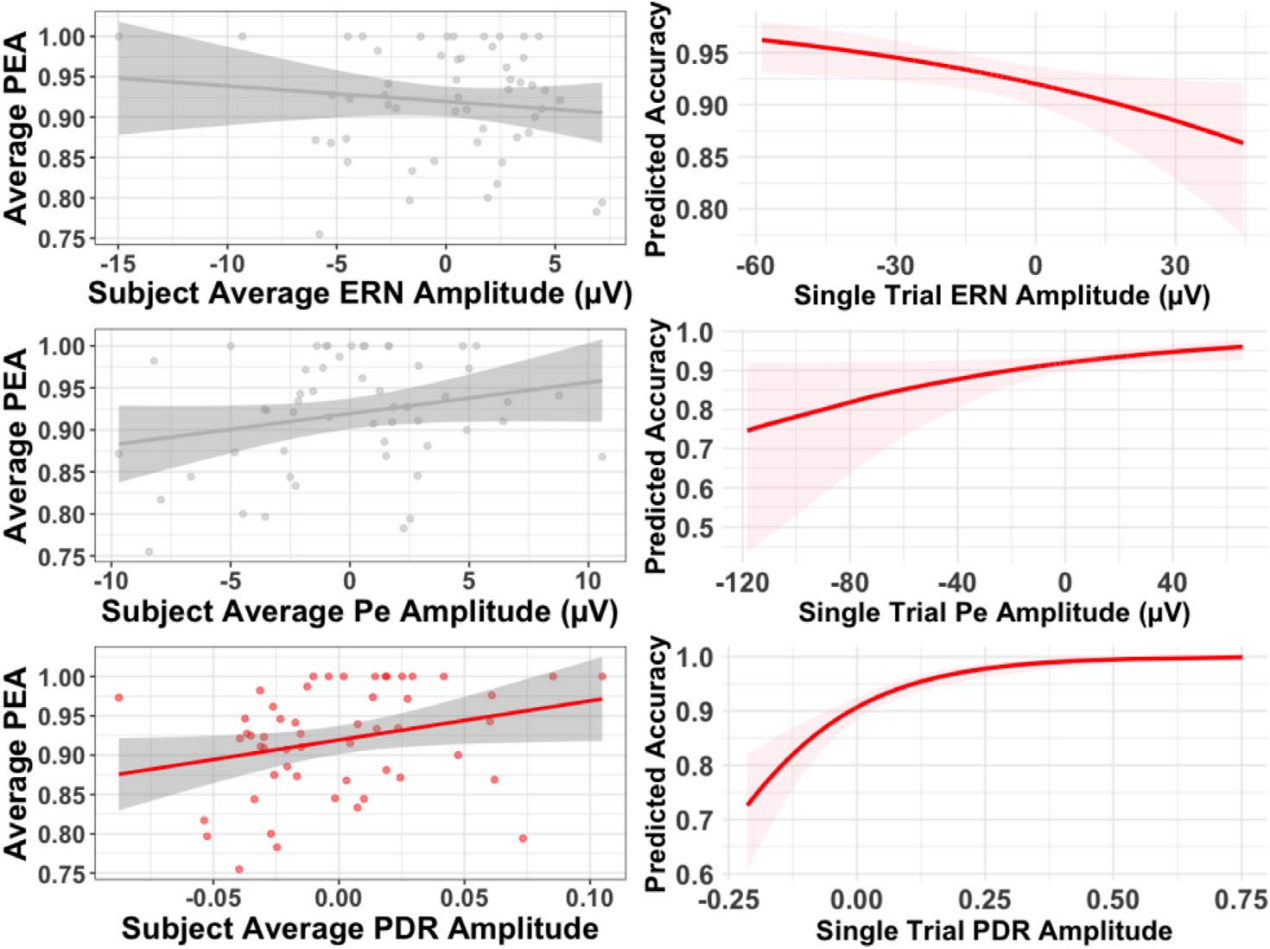
The left panel depicts subject‐level relationships between average ERN, Pe, and PDR amplitude and average post‐error accuracy for error trials only. Right panel depicts single‐trial relationships between subject‐mean centered Pe, ERN and PDR amplitude and accuracy on the following trial (for errors only). Estimate of the odds ratio is plotted for each predictor variable. For all subplots, red lines indicate a significant relationship.

We next explored both inter and intra‐individual relationships between our physiological error‐processing variables and post‐error accuracy. For both intra and inter‐individual models, we only included trials preceded by errors. First, we examined how ERN amplitude on a given error trial influenced accuracy on the following trial (intra‐individual relationships). We included ERN amplitude as a fixed‐effects predictor, subject as a random intercept, and accuracy on the following trial as the outcome. As we were only looking at error trials specifically, there was no interactive term included. The addition of ERN amplitude as a random slope across subjects did not improve the model (*p* > 0.05), so it was not included. We found a significant effect of ERN amplitude on accuracy such that as ERN amplitude increased (became more negative), the odds of getting the next trial correct increased (See Table 3; Figure 4). Interestingly, however, for inter‐individual analyses, we found no significant relationship between subject‐level ERN amplitude and average post‐error accuracy (See Table 4; Figure 4).

**TABLE 3:** Models for examining intra‐individual relationships between ERN, Pe, and PDR and robust post‐error accuracy (PEA), *N* = 53 subjects, 3225 observations (errors only).

Predictors	Odds ratios	CI	χ^2^	df	*p*
Model 1: ERN on PEA					
(Intercept)	11.51	8.73–15.17	—	—	—
ERN amplitude	0.99	0.98–1.00	5.80	1	0.02*
*Random effects*	*σ* ^2^	*τ*_00 (Subject)_	*τ*_11_	*ρ*_01_	
	3.29	0.50	—	—	
Model 2: Pe on PEA					
(Intercept)	11.37	8.62–14.99	—	—	—
Pe amplitude	1.01	1.00–1.02	4.59	1	0.04*
*Random effects*	*σ* ^2^	*τ*_00 (Subject)_	*τ*_11_	*ρ*_01_	
	3.29	0.47	—	—	
Model 3: PDR on PEA					
(Intercept)	9.73	7.48–12.64	—	—	—
PDR amplitude	436.67	32.89–5797.18	21.00	1	< 0.001***
*Random effects*	*σ* ^2^	*τ*_00 (Subject)_	*τ*_11 (Subject × PDR am)_	*ρ*_01 (Subject)_	
	3.29	0.40	13.77	0.23	

*= *p* < .05, *** = *p* < .001.

**TABLE 4:** Models for examining inter‐individual effects of ERN, Pe, and PDR amplitude on post‐error accuracy (PEA), *N* = 53 subjects, averages computed on error trials only.

Predictors	Beta estimates	SE	*t*	df	*p*
Model 1: ERN on PEA					
(Intercept)	0.91	0.01	—	—	—
ERN amplitude	−1.93 × 10^−3^	2.24 × 10^−3^	−0.86	51	0.39
Model 2: Pe on PEA					
(Intercept)	0.92	0.01	—	—	—
Pe amplitude	3.72 × 10^−3^	2.14 × 10^−3^	1.74	51	0.09
Model 3: PDR on PEA					
(Intercept)	0.92	0.01	—	—	—
PDR amplitude	0.50	0.24	2.08	51	0.04*

*= *p* < .05.

Next, we examined how Pe amplitude on a given error trial influenced accuracy on the following trial. We included Pe amplitude as a fixed‐effects predictor, subject as a random intercept, and accuracy on the following trial as the outcome. The addition of Pe amplitude as a random slope across subjects did not improve the model (*p* > 0.05), so it was not included. We found a significant effect of Pe amplitude on accuracy, such that as Pe amplitude increased (became more positive), the odds of getting the next trial correct also increased (See Table 3; Figure 4). Note that this effect was larger in magnitude than the ERN effect (though no formal statistical comparison was conducted). For inter‐individual analyses we found a marginal but non‐significant relationship between average Pe amplitude following errors and average PEA, such that as Pe amplitude increased, so did PEA (see Table 4; Figure 4).

Next, we examined how PDR amplitude on a given error trial influenced accuracy on the following trial. We included PDR amplitude as a fixed‐effects predictor, subject as a random intercept, and accuracy on the following trial as the output. The addition of PDR amplitude as a random slope across subjects improved model fit (χ^2^(2) = 7.15, *p* = 0.03), so it was included. We found a significant effect of PDR amplitude on accuracy such that as PDR amplitude increased, the odds of getting the next trial correct also increased (See Table 3; Figure 4). This effect was descriptively the largest in overall effect size of the three predictors (though note that no formal statistical comparison was conducted). For inter‐individual analyses, we also found a significant relationship between average PDR amplitude and average PEA, such that individuals with a larger PDR amplitude also showed greater PEA (see Table 4; Figure 4). In summary, all three predictor variables predicted accuracy on the next trial at the single‐trial level. PDR was the only predictor at the subject level that significantly predicted individual differences in PEA.

### 3.3 Relationships Between the ERN, Pe, and PDR

**FIGURE 5:**
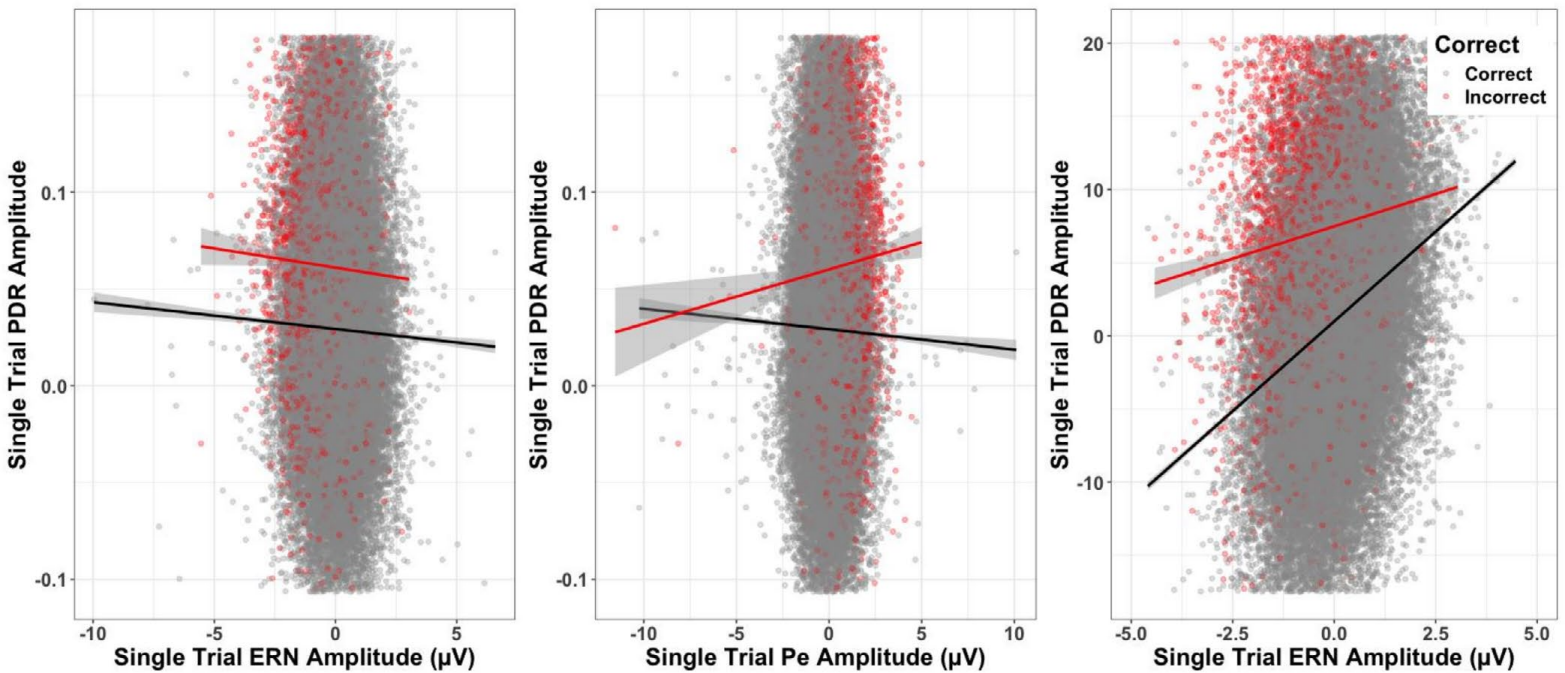
The left panel depicts single‐trial relationships between subject‐mean centered single trial ERN amplitude and pupillary amplitude for correct (gray/black), and incorrect (red) trials. Lines of best fit are drawn. Middle panel depicts single‐trial relationships between subject‐mean centered Pe amplitude and pupillary amplitude for correct (gray/black), and incorrect (red) trials. Lines of best fit are drawn. Right panel depicts single‐trial relationships between subject‐mean centered single trial ERN amplitude and Pe amplitude for correct (gray/black), and incorrect (red) trials. Y‐axes are set to ±2 SD of the *y* variable (Witt 2019).

Finally, we examined relationships between the ERN, Pe and PDR. First, we tested whether ERN predicted the PDR at the single‐trial level. Subject‐mean centered ERN amplitude, accuracy of the current trial (i.e., correct vs. incorrect), and their interaction were used as the fixed effects. The addition of accuracy (correct vs. incorrect) as a random slope (and its correlation with the intercept) across subjects resulted in a better fit (χ^2^(2) = 279.08, *p* < 0.001), so they were also included in the model. While there was no significant interaction, we did find a main effect of accuracy (See Table 5) such that incorrect trials elicited a larger PDR amplitude than correct trials, consistent with our prior reporting (LoTemplio, Silcox, et al. 2023). We also found a main effect of ERN amplitude (See Table 5) such that as ERN amplitude was more negative, pupillary amplitude increased (See Figure 5), for both correct and incorrect trials.

**TABLE 5:** Models for examining intra‐individual relationships between the ERN, Pe, and PDR.

Predictors	Beta estimates	SE	χ^2^	df	*p*
Model 1: ERN on pupillary outcomes					
(Intercept)	0.03	< 0.01	—	—	—
Accuracy	0.06	< 0.01	90.26	1	< 0.001***
ERN amplitude	−1.79 × 10^−4^	< 0.0001	6.52	1	0.01*
Accuracy × ERN amplitude	5.24 × 10^−4^	< 0.0001	0.19	1	0.67
*Random effects*	*σ* ^2^	*τ*_00 (Subject)_	*τ*_11 (Subject × Accuracy)_	*ρ*_01_	
	4.49 × 10^−3^	4.72 × 10^−4^	6.35 × 10^−4^	0.21_Subject_	
Model 2: Pe on pupillary outcomes					
(Intercept)	0.03	< 0.01	—	—	—
Accuracy	0.06	< 0.01	90.44	1	< 0.001***
Pe amplitude	−1.76 × 10^−4^	< 0.0001	0.31	1	0.58
Accuracy × Pe amplitude	2.18 × 10^−4^	< 0.0001	5.06	1	0.02*
*Random effects*	*σ* ^2^	*τ*_00 (Subject)_	*τ*_11 (Subject × Accuracy)_	*ρ*_01_	
	4.49 × 10^−3^	4.72 × 10^−4^	6.33 × 10^−4^	0.21_Subject_	
Model 3: ERN on Pe outcomes					
(Intercept)	0.88	0.30	—	—	—
Accuracy	10.27	0.62	98.84	1	< 0.001***
ERN amplitude	0.33	< 0.01	1414.40	1	< 0.001***
Accuracy × ERN amplitude	−0.08	0.02	30.77	1	< 0.001**
*Random effects*	*σ* ^2^	*τ*_00 (Subject)_	*τ*_11 (Subject × Accuracy)_	*ρ*_01_	
	70.97	4.58	18.03	−0.14_Subject_	

*Note: N* = 53 subjects, 54,212 observations. Accuracy was dummy‐coded such that 0 represented a correct response and 1 represented an error response. Therefore, accuracy main effects are errors compared to correct responses.* = *p* < .05, ** = *p* < .01, *** = *p* < .001.

Using the same model structure as above, we examined how subject‐mean centered single‐trial Pe amplitude affected PDR amplitude in a separate model. The addition of a random slope of accuracy (and its correlation with the intercept) improved fit (χ^2^(2) = 274.19, *p* < 0.001), so they were included in the model. There was a main effect of accuracy, such that Pe amplitude was larger on error trials. However, these effects were qualified by a significant interaction of Pe amplitude and accuracy (See Table 5; Figure 5). We decomposed the interaction using *emmeans()* finding that on correct trials, a larger single‐trial Pe amplitude predicted smaller PDR amplitudes (simple slope estimate = −1.78 × 10^−4^; 95% CI = [−2.41 × 10^−4^, −1.11 × 10^−4^]). On incorrect trials, a larger single trial Pe amplitude did not significantly predict PDR amplitudes (simple slope estimate = 1.06 × 10^−4^; 95% CI = [−1.31 × 10^−4^, 3.43 × 10^−4^]).

In the same model structure as above, we also examined whether single‐trial ERN amplitude predicted single‐trial Pe amplitude. The addition of accuracy (correct vs. incorrect) as a random slope (and its correlation with the intercept) across subjects resulted in a better fit (χ^2^(2) = 555.18, *p* < 0.001), so they were included in the model. We found significant main effects of both accuracy and subject‐mean centered ERN amplitude on Pe amplitude (See Table 5). However, this was qualified by a significant interaction between the two terms (See Table 5; Figure 5). We decomposed the interaction, finding that on correct trials, a larger (more negative) single‐trial ERN amplitude predicted smaller Pe amplitudes (simple slope estimate = 0.33; 95% CI = [0.32, 0.34]). On incorrect trials, a larger (more negative) single‐trial ERN amplitude predicted smaller Pe amplitudes, though not as strongly as on correct trials (simple slope estimate = 0.24; 95% CI = [0.22, 0.27]).

**FIGURE 6:**
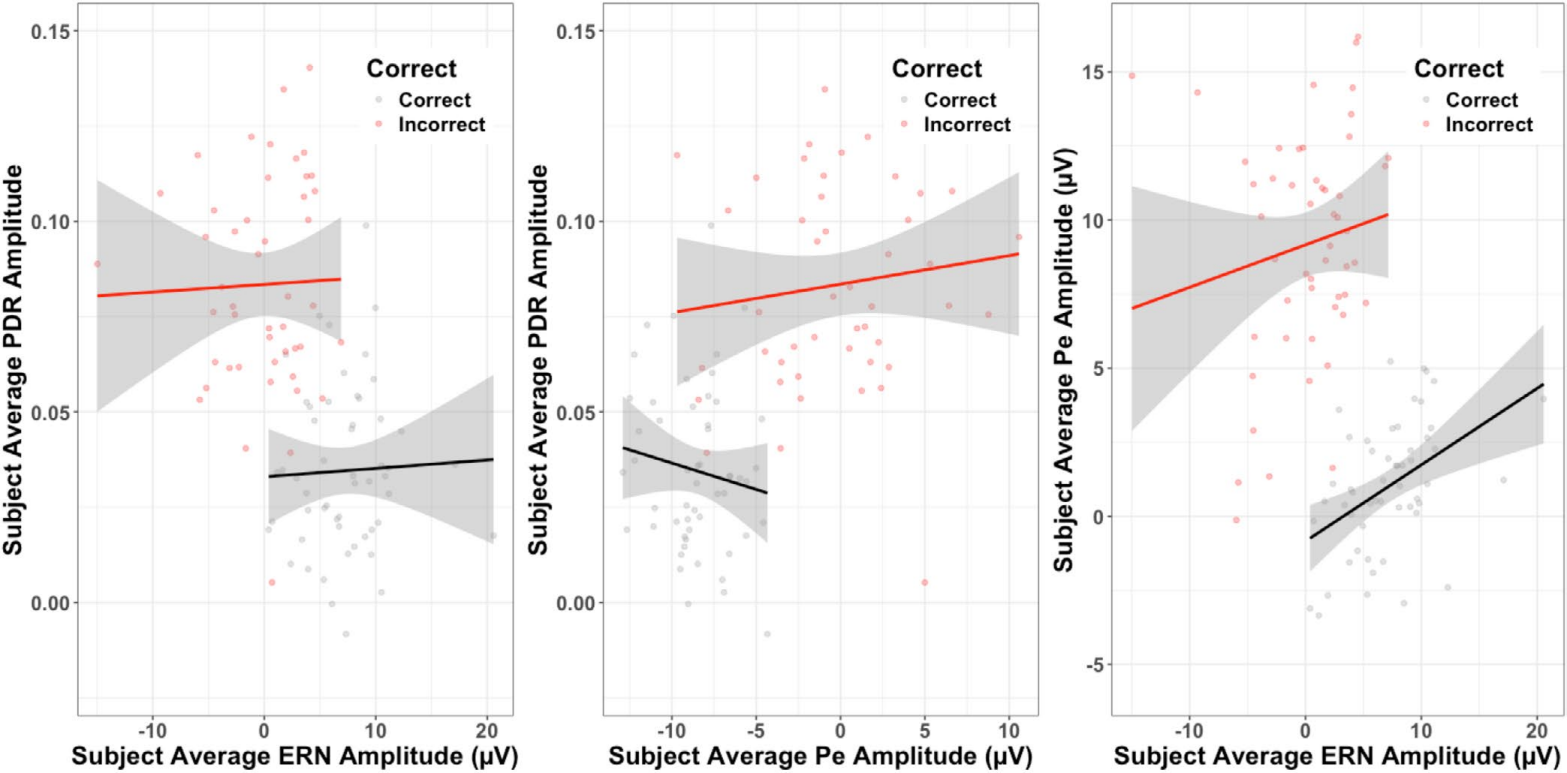
The left panel depicts single‐trial relationships between subject‐level ERN amplitude and average pupillary amplitude for correct (gray/black), and incorrect (red) trials. Lines of best fit are drawn. Middle panel depicts subject‐level relationships between average Pe amplitude and pupillary amplitude for correct (gray/black), and incorrect (red) trials. Lines of best fit are drawn. Right panel depicts subject‐level relationships between average ERN amplitude and Pe amplitude. Lines of best fit are drawn. Y‐axes are set to ±2 SD of the *y* variable (Witt 2019).

We also examined the relationships between the ERN, the Pe, and the PDR at the subject‐level. The inclusion of accuracy as a random slope did not improve model fit (*p* > 0.05), so no random slopes were included. We found that there was no significant main effect of subject‐level ERN amplitude on PDR amplitude, nor an interaction with accuracy (*p* > 0.05; See Table 6). There was a main effect of accuracy, such that, as in the intra‐individual analyses, PDR amplitude was on average larger for incorrect trials compared to correct (See Table 2; Figure 6). However, we found no main effect of subject‐level Pe amplitude or ERN amplitude on PDR amplitude, nor an interaction of either of these variables with accuracy on PDR amplitude (*p* > 0.05; See Table 6). Finally, we also examined how subject‐level ERN amplitude predicted Pe amplitude. The inclusion of accuracy as a random slope did not improve model fit (*p* > 0.05), so no random slopes were included. We found a significant effect of accuracy, such that Pe amplitude was larger on incorrect trials (See Table 6; Figure 6). However, there were no main effects of ERN amplitude, nor an interaction between ERN amplitude and accuracy on Pe amplitude (*p* > 0.05).

**TABLE 6:** Models for examining inter‐individual relationships between the ERN, Pe and PDR.

Predictors	Beta estimates	SE	χ^2^	df	*p*
Model 1: ERN on pupillary outcomes					
(Intercept)	0.04	< 0.01	—	—	—
Accuracy	0.05	0.01	39.78	1	< 0.001**
ERN amplitude	−8.24 × 10^−4^	< 0.001	0.21	1	0.65
Accuracy × ERN amplitude	0.001	0.001	0.96	1	0.33
*Random effects*	*σ* ^2^	*τ*_00 (Subject)_	*τ*_11 (Subject × Accuracy)_	*ρ*_01_	
	3.66 × 10^−4^	6.09 × 10^−4^	—	—	
Model 2: Pe on pupillary outcomes					
(Intercept)	0.03	< 0.01	—	—	—
Accuracy	0.06	0.01	33.16	1	< 0.001**
Pe amplitude	6.30 × 10^−4^	1.54 × 10^−3^	0.20	1	0.66
Accuracy × Pe amplitude	2.05 × 10^−3^	< 0.001	1.68	1	0.19
*Random effects*	*σ* ^2^	*τ*_00 (Subject)_	*τ*_11 (Subject × Accuracy)_	*ρ*_01_	
	3.57 × 10^−4^	5.92 × 10^−4^	—	—	
Model 3: ERN on Pe outcomes					
(Intercept)	−0.50	0.99	—	—	—
Accuracy	10.07	1.06	66.17	1	< 0.001
ERN amplitude	0.21	0.12	2.21	1	0.14
Accuracy × ERN amplitude	−0.16	0.16	0.92	1	0.34
*Random effects*	*σ* ^2^	*τ*_00 (Subject)_	*τ*_11 (Subject × Accuracy)_	*ρ*_01_	
	9.88	1.42	—	—	

*Note: N* = 53 subjects, 106 observations. Accuracy was dummy‐coded such that 0 represented a correct response, and 1 represented an error response. Therefore, accuracy main effects are errors compared to correct responses.** = *p* < .01.

In summary, we only found reliable relationships between the ERN and PDR and the Pe and PDR at the single‐trial level. At the single‐trial level, ERN amplitude negatively predicted PDR amplitude, regardless of the correctness of the trial. Meanwhile, Pe amplitude differentially predicted PDR amplitude for correct trials compared to incorrect trials. ERN amplitude also predicted Pe amplitude differentially, with a strong relationship between ERN amplitude and Pe amplitude on correct trials and a weaker but still significant relationship on incorrect trials.

### 3.4 Exploratory Analyses

While we initially hypothesized and ran the models with each predictor variable separately, we noticed that all three predictor variables were significantly related to post‐error accuracy at the single‐trial level. We decided to conduct an exploratory analysis that included all predictors in the model to see if any of the predictors explained unique variance in post‐error accuracy after accounting for the effects of the other predictors. For these analyses, we subject‐mean standardized^3^ the predictor variables to be able to qualitatively compare relative effect sizes across the three variables. To do this, in addition to subject‐mean centering, we divided each predictor value by the subject's standard deviation for that value. We found that the results remained constant with the inclusion of all predictors in a single model, with all three physiological variables significantly predicting unique variance in post‐error accuracy, but with PDR amplitude showing the overall largest effect (see Table 7).

**TABLE 7:** Results from exploratory analysis including all predictor terms for single‐trial post‐error accuracy.

Model 1: PDR, ERN, and Pe amplitude on PEA			
Predictors	Odds ratios	CI	*p*
(Intercept)	7.85	5.91–10.44	—
PDR amplitude	1.35	1.21–1.50	< 0.001***
ERN amplitude	0.84	0.76–0.94	< 0.01**
Pe amplitude	1.15	1.04–1.28	< 0.01**
Random effects			
*σ* ^2^	3.29		
*τ*_00 Subject_	0.39		
ICC	0.11		
*N*_Subject_	53		
Observations	3225		
Marginal *R* ^2^/Conditional *R* ^2^	0.048/0.150		

*Note:* Odds ratios are standardized.** = *p* < .01, *** = *p* < .001.

Given that both Pe amplitude and ERN amplitude also predicted PDR amplitude, we similarly ran an exploratory regression with both predictors in the model to see if one explained the variance of the other. We found that even when including both predictors in the model, results held, suggesting that both the ERN and Pe predict unique variance in PDR amplitude.

Additionally, we were intrigued to see that only subject‐level ERN amplitude predicted post‐error slowing. Given the well‐established relationship between subject‐level ERN amplitude and anxiety (e.g., Hajcak et al. 2003; Meyer 2017; Weinberg, Klein, and Hajcak 2012; Xiao et al. 2011), we wondered whether differences in trait‐level anxiety could also contribute to increased cautionary slowing following errors. Therefore, we ran an exploratory inter‐individual analysis of ERN amplitude on post‐error slowing with trait‐level anxiety (the Penn State Worry Questionnaire, PSWQ; Meyer et al. 1990) as an interactive variable and main effect. We found that there was no significant relationship between trait worry and post‐error slowing, nor was there a significant interaction between trait worry and ERN amplitude, nor did trait worry's inclusion improve model fit (*p* > 0.05 for both). In an attempt to replicate prior work showing a robust relationship between trait anxiety and ERN amplitude, we also ran an exploratory regression with the PSWQ as a subject‐level predictor variable and ERN amplitude as an outcome variable. Interestingly, we did not replicate prior work, finding no significant relationship between these two variables.

## 4 Discussion

The goal of the current work was to characterize how each index of error‐processing predicted both error correction and slowing, as well as to understand the relationship among the three variables. Though there has been much discussion around the appropriate level of analysis (e.g., single trial vs. subject level) and the appropriate metric (e.g., post‐error slowing vs. post‐error accuracy) to understand relationships between the ERN and post‐error behavior adjustment (for review, see LoTemplio, Lopes, et al. 2023), to our knowledge, no prior work has directly compared both levels of analysis and metrics in the same study. It is important to understand this, as this analysis would provide insight into whether inter‐individual analyses show the same pattern of results as intra‐individual analyses. The results could inform analytical choices for researchers in the future who are interested in post‐error behavior adjustment. Further, no work has also additionally and simultaneously examined relationships between additional markers of error‐processing (the Pe and the PDR) at multiple levels of analysis within the same study. This is an important first step to understanding the extent to which these components of error‐processing are distinct versus share common variance and, potentially, underlying neural circuits. Our study aimed to bridge these gaps and clarify relationships among neural indices of error‐processing and behavior adjustment.

In terms of behavior, all three psychophysiological variables predicted unique variance in post‐error accuracy on the single‐trial level, with the largest effect size being for PDR amplitude in predicting post‐error accuracy. At the subject level, only average PDR amplitude predicted average post‐error accuracy. For post‐error slowing, only single‐trial Pe amplitude predicted reaction time on the following trial. Meanwhile, at the subject level, only average ERN amplitude predicted average robust PES, such that larger average ERN amplitudes (more negative) predicted greater slowing. This relationship sustained when including trait‐level anxiety in the model, as measured by the PSWQ. We also found that, at the single‐trial level, both Pe and ERN amplitude predicted PDR amplitude, but that this relationship was only present between the Pe and PDR for correct trials. We also found that ERN amplitude predicted Pe amplitude, but that this relationship was stronger on correct trials than incorrect trials (although still significant on incorrect trials). Below, we discuss these findings in more detail as well as their implications for theories of error‐processing.

### 4.1 Post‐Error Slowing

We observed distinct patterns of physiological predictors of post‐error slowing depending on if we analyzed data at the intra or inter‐individual level. At the inter‐individual (subject‐average) level, we found that more negative ERN amplitudes predicted greater slowing for incorrect trials. However, we found no relationship between the ERN and robust post‐error reaction time at the single‐trial level, despite observing a relationship at the single‐subject level. These results add to the growing inconsistency in linking ERN amplitude to post‐error slowing (for review see LoTemplio, Lopes, et al. 2023), though it is worth noting again that post‐error slowing may not necessarily be the best measure of adaptive behavior adjustment (Schroder et al. 2020).

Given this dissociation, it is possible that these results emerged at the inter‐individual level due to a third, confounding subject‐level variable. However, when we examined whether trait anxiety might account for the relationship between ERN amplitude and slowing following an error, no significant relationship was found. It is possible that such a relationship was not found because the measurement used, the PSWQ, is thought to specifically account for the anxious apprehension dimension of anxiety (for review see Sharp et al. 2015). Therefore, it is still possible that this relationship could be explained by a more generalized measure of anxiety, such as the GAD‐7 or the MASQ‐AA (e.g., Spitzer et al. 2006; Watson et al. 1995). It is also entirely possible that this relationship is *not* confounded by a third variable such as anxiety. Future work is necessary to understand this relationship between the ERN and average post‐error slowing.

The only measure that significantly predicted post‐error reaction time at the single‐trial level was Pe amplitude, such that larger Pe amplitudes to incorrect trials indicated slower reaction times on the following trial. This is consistent with accounts that suggest that the Pe represents a build‐to‐threshold evidence accumulation process to recognize errors (e.g., Steinhauser and Yeung 2010). Prior work has also extensively posited that larger orienting responses in the brain may be linked to greater slowing on following trials (e.g., Wessel 2018). Therefore, it is also possible that a larger Pe might either represent or trigger a larger (re‐)orienting response that then causes slower reaction times. However, further work is needed to understand the extent to which the Pe is related to the orienting response.

We found no relationship between error‐related PDR amplitude and post‐error slowing, at either the subject level or single‐trial level. This is somewhat surprising, as past work has linked stimulus‐related PDR amplitude to reaction time (e.g., LoTemplio et al. 2020), and at least one study has linked specifically error‐related PDR amplitude to post‐error slowing (e.g., Murphy et al. 2016). However, Murphy et al. (2016) used an unusually long ISI (6000 ms). Therefore, it is possible that our response–stimulus interval (RSI), which was 1500 ms, was not long enough and forced participants to respond before the full error recognition and adjustment process was complete.

### 4.2 Post‐Error Accuracy

We found that all three physiological indices of error‐processing predicted post‐error accuracy at the single‐trial level, but that only Pe amplitude additionally predicted post‐error accuracy at the subject level. Prior work has long suggested that intra‐individual analyses of post‐error slowing may be a more appropriate way of measuring relationships between the ERN and behavior adjustment (for review see LoTemplio, Lopes, et al. 2023). However, much of this work specifically examined post‐error slowing, with little work examining single‐trial relationships between the ERN and post‐error *accuracy*, which is usually a more direct measure of behavior adjustment (Schroder et al. 2020). Even less work has examined relationships between single‐trial measures of Pe and PDR amplitude and accuracy. The present study extends this literature, demonstrating a robust relationship between all three indices of error‐processing and behavior adjustment. Furthermore, it appears that each of the physiological variables still predicts unique variance in post‐error accuracy when the other physiological variables were also included in the model, suggesting that the ERN, the Pe, and the PDR each reflect distinct physiological contributors to post‐error accuracy. Our results indicate that each physiological response can be used to predict post‐error accuracy, but only at the single‐trial level. Therefore, we recommend that researchers interested in trial‐to‐trial brain‐behavior relationships use this level of analysis in future work. Future work is also needed to further clarify the unique contributions of each physiological outcome to post‐error accuracy.

### 4.3 Relationships Among ERN, Pe, and PDR

Consistent with our hypothesis, we found that both the ERN and the Pe predicted PDR amplitude, but only at the single‐trial level. However, the Pe differentially predicted PDR amplitude for correct trials vs. incorrect trials. This interesting association suggests that the ERN and Pe may be accounting for different portions of the variance in PDR amplitude. Indeed, exploratory analyses including both predictors in the model simultaneously controlling for the other did not change results, consistent with this interpretation.

Given that there was a main effect of ERN amplitude on the PDR that was not qualified by an interaction with accuracy (i.e., correct vs. incorrect trials), it seems that the relationship may be due to fluctuations in trial‐to‐trial overall arousal levels, irrespective of error commission. It's important to note that this finding suggests that there is no differential *error* effect (as one might measure in a difference score average ERP). However, there is evidence suggesting that both ERN amplitude and PDR amplitude are influenced by attention to task—for example, dual‐tasking decreases ERN amplitude (e.g., Klawohn et al. 2016; Pailing and Segalowitz 2004), and also decreases PDR amplitude (LoTemplio, Silcox, et al. 2023). Further, ERN amplitude decreases when participants are mentally and attentionally fatigued (e.g., Boksem et al. 2006; Kato et al. 2009; Xiao et al. 2015). Therefore, it is possible that attention to task, which can fluctuate on both a state level and between individuals, affects both ERN amplitude and PDR amplitude separately. Thus, future work is needed to assess whether real‐time attention to task explains these relationships, perhaps through attention probing experiments. It would also be useful to assess the extent to which attention to task differentially affects error vs. correct trials. Finally, given the above links to attention/fatigue, and given the link to the LC‐NE system with both the ERN (Riba et al. 2005) and the PDR (Joshi and Gold 2020), it is possible that the shared variance among the components regardless of trial type (correct vs. incorrect) has more to do with overall state variation in arousal.

However, the differential effects of trial accuracy on relationships between the Pe and the PDR suggest unique effects for correct trials *specifically*. In other words, there was also no differential *error* effect. This was surprising to us and somewhat inconsistent with the notion that the Pe and the PDR both reflect phasic LC‐NE responses to errors in the brain. Therefore, it is possible that the Pe and the PDR reflect separable portions of the error‐processing stream, with the Pe being more closely aligned with error awareness rather than the orienting response that the PDR is thought to reflect (among other things). Further work is needed to understand if the Pe more closely aligns with error awareness, an LC‐NE driven orienting response (Gabay et al. 2011), or both.

Interestingly, we saw that ERN amplitude significantly predicted Pe amplitude, especially so for correct trials, but only at the single‐trial level. There were no significant relationships between these variables at the subject level. This is particularly notable when considering recent work has documented divergent validity among the ERN and Pe (e.g., Clayson, Mcdonald, et al. 2025; Clayson, Baldwin, and Larson 2025; Riesel et al. 2013). This discrepancy could again be because prior work uses subject‐level averages of ERN and Pe amplitude. As this previous research was examining psychometric convergence vs. divergence of ERP components, the use of subject‐level averages is obviously warranted. However, interestingly, the trial‐to‐trial dynamics of the ERN and Pe appear to be linked, even when subject‐mean centering to isolate within‐subjects variability from between‐subjects differences. It would be interesting for future work to track the dynamics between these variables over time to better understand how time on task and potential arousal/engagement levels could affect these relationships.

### 4.4 Summary and Future Directions

Interestingly, the Pe appeared to predict both post‐error slowing and post‐error accuracy at the single‐trial level (and post‐error accuracy at the subject level), as well as the error‐related PDR. This perhaps suggests that the Pe, rather than the ERN, might be a better generalized marker for adaptive behavior adjustment. However, the ERN still predicted behavior adjustment in terms of accuracy, even when accounting for Pe in the model. Thus, each physiological variable appears to uniquely influence behavior, rather than sequentially influencing behavior. For example, a possible pathway could be that the ERN triggers cascading processes that affect Pe amplitude, which in turn affects RT and perhaps adjusts arousal as measured by pupillary amplitude. If the ERN is a marker of error detection, and the Pe is a marker of error awareness and/or an orienting response to errors, it would stand to reason that both may influence accuracy on the next trial. Further work is necessary to examine the unique contributions of the ERN and Pe to post‐error accuracy.

It is worth noting that the error‐related PDR was the strongest predictor of post‐error accuracy. There is also a strong history of evidence linking pupil size to orienting responses (e.g., Gabay et al. 2011; Mathôt et al. 2013). The PDR is also directly related to phasic LC‐NE activity (for review see Joshi and Gold 2020) that is largest when arousal is at optimal levels on the Yerkes‐Dodson curve (Yerkes and Dodson 1908). Therefore, our findings that larger PDR amplitude leads to greater post‐error accuracy are consistent with this account as greater amplitudes likely indicate optimal arousal levels for high task‐related performance.

Both the ERN and Pe appear to predict unique variance in PDR amplitude, but the relationship between the Pe and PDR depends on accuracy of trial (correct only). This seems to suggest that, especially for error trials, the Pe and PDR may represent separable components of the error‐processing pipeline (e.g., error awareness vs. orienting, respectively). An interesting future study to determine any potential overlap between the Pe and the orienting response could manipulate ISI, examining whether the Pe and PDR are more related at shorter ISIs that may elicit orienting responses (Wessel 2018).

On the other hand, the link between the ERN and the PDR is not dependent on trial accuracy, perhaps indicating the ERN and PDR share variance relating more so to general arousal levels—perhaps related to LC‐NE activity. Additional work is needed to further assess this. Specifically, studies concurrently recording fMRI and EEG may provide additional clarity, as well as studies that aim to specifically influence arousal. For example, prior work has successfully increased LC‐NE activity and subsequent arousal through the use of isometric hand grip manipulations (e.g., Nielsen and Mather 2015). Therefore, future work could leverage this manipulation to understand whether it similarly affects both the ERN and PDR. Finally, recent work has also determined that pre‐trial alpha asymmetry activity related to a distracting stimulus also predicted ERN amplitude (Maier and Steinhauser 2025). The authors suggest that this could trigger attentional adjustments, which are also known to be linked to pupil diameter (Van Den Brink et al. 2016). Thus, future research should examine how dual‐task and other attentional manipulations affect each outcome.

Finally, this work is limited in that the relationships explored are correlational in nature. Future work could use experimental methods, such as the hand grip manipulation, to further understand the relationship between the PDR and both the ERN and Pe. Similarly, a recent paradigm was able to experimentally eliminate the elicitation of the ERN without eliminating the ERN (Di Gregorio et al. 2018; Dumsky et al. 2025). Future work could use this paradigm to understand how the elimination of the ERN or the Pe affected the PDR to better understand potential causal links. Additionally, a recent paper found that time‐on‐task can affect the ERN and Pe amplitudes (Clayson, Baldwin, and Larson 2025). While we do not anticipate this being a major driver of data patterns within the current data set, as participants were only “on task” for 1–2 min at a time, future work should examine whether relationships among these variables and behavior shift according to time on task.

## 5 Conclusions

In summary, the most striking finding of the current study was that each physiological error‐processing measure—the ERN, Pe, and PDR—were successfully and uniquely linked to post‐error accuracy at the single‐trial level. Prior work has suggested that post‐error accuracy may be a better measure of adaptive post‐error behavior adjustment than post‐error slowing (Schroder et al. 2020). Interestingly, only the PDR predicted post‐error accuracy at the subject level, and only the Pe predicted post‐error slowing at the single‐trial level. The Pe and PDR have received considerably less attention than the ERN, and yet in our sample, these physiological indices of error‐processing were more consistently and more robustly linked to post‐error behavioral corrections than the ERN. The Pe also showed a stronger and more distinctive relationship with the error‐related PDR than the ERN did, in that it was moderated by error commission, whereas the ERN was not. Thus, further work is needed to disentangle the influence that the Pe has on both downstream adjustment of arousal (as measured by the PDR) and subsequent corrective behavior.

Importantly, our results also demonstrate the importance of examining relationships among psychophysiological variables at multiple levels of analyses. Researchers have long cautioned against using only subject‐level analyses to determine brain‐behavior relationships (e.g., see LoTemplio et al. 2021; Weinberg, Riesel, et al. 2012). Our results reiterate this point in showing a dissociation between intra‐ and inter‐individual relationships. Finally, the physiological measures of error‐processing were overall less successfully linked to post‐error slowing. This adds to the growing inconsistency in linking the ERN to post‐error slowing and to suggestions that post‐error slowing may not inherently be adaptive (e.g., Wessel 2018). Collectively, the findings from the current study underscore the necessity of characterizing brain‐behavior relationships at multiple levels of analysis to accurately characterize the cooperative dynamics of error‐processing components and their influence on subsequent behavioral adjustment.

## Author Contributions

**Sara LoTemplio:** conceptualization, investigation, writing – original draft, methodology, visualization, writing – review and editing, validation, software, formal analysis, project administration, data curation. **Jack Silcox:** software, formal analysis, writing – review and editing, writing – original draft, methodology. **David L. Strayer:** writing – original draft, writing – review and editing, supervision. **Brennan R. Payne:** supervision, writing – original draft, writing – review and editing, resources.

## Funding

This work was supported, in part, by NSF GRFP awarded to SL (DGE‐1656518).

## Conflicts of Interest

The authors declare no conflicts of interest.

## Data Availability

Pre‐processed aggregate de‐identified data are available at this link: https://osf.io/z3de2/overview?view_only=6c224254fcfd4cdbaec1aabf9f73756d.

## Endnotes

^1^
It should be noted that the intra‐individual analyses referred to here do not account for scenarios in which trait‐level variables influence the intra‐individual relationships *themselves* between predictors and outcomes. For example, we might imagine that the intra‐individual relationship between number of cups of coffee that an individual has consumed in a day and their positive affect that day could vary depending on their anxiety‐ with the coffee consumption to positive affect ratio being positive in low anxiety individuals, and being negative in high anxiety individuals.

^2^
To ensure that our working memory manipulation did not fundamentally change relationships between our variables of interest (e.g., that the relationships between the PDR and ERN was not stronger or weaker in the high working memory condition vs. low working memory), we ran likelihood ratio tests for model comparisons with working memory manipulations and their interactions with fixed effects included in each model vs. not. The addition of working memory did not improve model fit (all *p* > 0.05) for any of the models, and so it was not included.

^3^
Note that at this point we also explored running the models listed in Section 3.1, 3.2, and 3.3 with subject‐level standardized variables (i.e., subtracting the subject‐level mean and dividing by the subject‐level SD). The pattern and direction of results remained the same, but it is worth noting that both the interactive effect for the Pe and Accuracy on PDR and the Pe on PES at the intra‐individual level shrunk to the point of no longer being significant at the *p* < 0.05 level. This could be because of larger differences in variability in Pe amplitude among subjects (compared to other components) that are driving effects. These differences would then necessarily shrink when standardizing variance. Given that this type of variability may be due to true variability within individuals, we elected to stay with our initial analysis plan. However, for full transparency, the results of these supplemental analyses are available at https://osf.io/z3de2/overview?view_only=6c224254fcfd4cdbaec1aabf9f73756d.
